# Pregnancy-associated thrombotic thrombocytopenic purpura complicated by Sjögren’s syndrome and non-neutralising antibodies to ADAMTS13: a case report

**DOI:** 10.1186/s12884-021-04167-9

**Published:** 2021-12-03

**Authors:** Lu Zhou, Yu Zhu, Miao Jiang, Jian Su, Xiaofan Liu, Yizhi Jiang, Hui Mu, Jie Yin, Li Yang, Haiyan Liu, Weidong Pan, Min Su, Hong Liu

**Affiliations:** 1 Hematology department, Affiliated Hospital of Nantong University, No 20 Xisi Road, Nantong, 226001 Jiangsu China; 2 Obstetrics and Gynecology Department, Affiliated Hospital of Nantong University, No 20 Xisi Road, Nantong, 226001 Jiangsu China; 3 Key Laboratory of Thrombosis & Hemostasis of Ministry of Health, Jiangsu Institute of Hematology, The First Affiliated Hospital of Soochow University, Suzhou, China; 4 State Key Laboratory of Experimental Hematology, Key Laboratory of Gene Therapy of Blood Diseases, Institute of Hematology and Blood Disease Hospital, Chinese Academy of Medical Sciences & Peking Union Medical College, Tianjin, China; 5 Hematology department, The First Affiliated Hospital of Wannan Medical College, Wuhu, China

**Keywords:** Thrombotic thrombocytopenic purpura, Pregnancy, Rituximab, Sjögren’s syndrome

## Abstract

**Background:**

Thrombotic thrombocytopenic purpura (TTP) is a severe and life-threatening disease. Given its heterogeneous clinical presentation, the phenotype of TTP during pregnancy and its management have not been well documented.

**Case presentation:**

We report here a 25-year-old woman, G1P0 at 36 weeks gestation, who developed severe thrombocytopenia and anemia. She was performed an emergent caesarean section 1 day after admission because of multiple organ failure. As ADAMTS 13 enzyme activity of the patient was 0% and antibodies were identified by enzyme-linked immunosorbent assay, she was diagnosed as acquired thrombotic thrombocytopenic purpura (aTTP). Furthermore, asymptomatic primary Sjögren’s syndrome was incidentally diagnosed on screening. After treatment with rituximab in addition to PEX and steroids, the activity of the ADAMTS 13 enzyme increased significantly from 0 to 100%.

**Conclusions:**

To the best of our knowledge, this is the first case report of concomitant TTP and asymptomatic Sjögren’s syndrome in a pregnant woman. It highlights the association between pregnancy, autoimmune disease, and TTP. It also emphasizes the importance of an enzyme-linked immunosorbent assay in the diagnosis and rituximab in the treatment of patients with acquired TTP.

**Supplementary Information:**

The online version contains supplementary material available at 10.1186/s12884-021-04167-9.

## Background

Thrombotic thrombocytopenic purpura (TTP) is a rare life-threatening disease with an untreated mortality rate of 90% [1]. TTP is characterised by extensive platelet thrombus in the microvasculature [2], thrombocytopenia, mechanical haemolysis, injury, and dysfunction of involved tissues and organs [3]. Typical clinical manifestations of TTP include “thrombocytopenia, microangiopathy haemolytic anaemia (MAHA), neuropsychiatric symptoms, renal function damage and fever” [4]. According to the Oklahoma TTP-HUS Registry, 70% of cases of TTP occur in women, 45% of whom are of child-bearing age [5]. Moreover, TTP occurs in one out of 25,000–100,000 pregnancies, mostly in the late third trimester or during the puerperium [6].

Due to its heterogeneous clinical presentation, the phenotype of TTP during pregnancy and its management have not been well documented [6, 7]. Pregnancy-associated TTP can be divided into congenital TTP (cTTP) and acquired TTP (aTTP) according to the patient’s genetic background and antibody detection. One study showed that 66% of women presenting with acute TTP during pregnancy or in the immediate postpartum period had late-onset previously undiagnosed congenital disease [6]. Pregnancy-related aTTP may be associated with autoimmune disease [8]. There have been several reports of aTTP presenting secondary to a connective tissue disease, such as systemic lupus erythematosus, mixed connective tissue disease, rheumatoid arthritis, scleroderma, or dermatomyositis. Compared to other connective tissue diseases, primary Sjögren’s syndrome (pSS) combined with TTP is quite rare [9].

Most patients with aTTP harbour ADAMTS13 inhibitors, while 11.5–17% of patients have non-neutralising antibodies [10]. Non-neutralising antibodies can only be detected by enzyme-linked immunosorbent assay-based detection methods [11]. We have encountered a case of pregnancy-associated TTP complicated by pSS with non-neutralising antibodies that was successfully treated with rituximab in addition to plasma exchange and pulse corticosteroid therapy.

## Case presentation

A 25-year-old woman who was in week 36 of her first pregnancy was admitted to our obstetrics department on October 18, 2019. She complained of bleeding from the sclera of her left eye, nose, and gingiva and reported dyspnoea. She also had a 2-day history of diarrhoea and blurred vision in the left eye. There was no medical history or family history of abnormal bleeding. The foetal heart rate was normal (140 beats per minute) and there were no uterine contractions. Obstetric ultrasonography revealed that the foetus had an appropriate gestational age. An initial complete blood cell analysis indicated a platelet count of 7.0 × 10^9^/L, a haemoglobin level of 66 g/L, a reticulocyte count of 5.71%, and a white blood cell count within the normal range. The unbound bilirubin level was elevated to 57.83 μmol/L and the serum lactate dehydrogenase (LDH) level to 4886 U/L. Direct and indirect Coombs tests were negative. Anti-SS-A/60KD (+), anti-SS-A/52KD (++), and anti-nuclear antibody (ANA) (+) were detected and anti-double-stranded DNA was negative on regular screening. TTP was suspected. However, she had no neurological symptoms or fever. No schistocytes were identified in her blood smear. Given that her blood pressure was increased to 140/80 mmHg, her renal function tests showed slight proteinuria (+++), and her aspartate aminotransferase (AST) was increased to 52 U/L, a diagnosis of pregnancy-related thrombotic microangiopathy was made. However, in view of the difficulty in distinguishing between TTP, HELLP (*H*aemolysis *E*levated *L*iver enzymes *L*ow *P*latelets) syndrome, pre-eclampsia, and haemolytic uraemic syndrome, an ADAMTS13 test was requested.

**Fig. 1:**
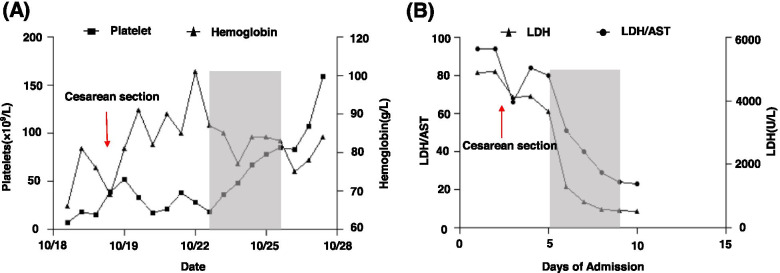
Peripheral blood examination during the course of the disease. The gray area represents the period of plasma exchange. (**A**) Platelet, hemoglobin level and treatment sessions during the course of the disease. (**B**) LDH and LDH/AST level during the course of the disease

While waiting for the ADAMTS13 test result, the patient progressed to hypoxaemia, bilateral hydrothorax, and heart failure. Given that the patient was nearly full-term and had a significantly decreased platelet count, a caesarean section was performed immediately. This resulted in delivery of a healthy female baby weighing 2100 g (with Apgar scores of 9 after 1 min and 10 after 5 min) who was transferred to the neonatal care unit. The operation went well, and 100 mL of bloody ascites was noted during surgery. However, the patient’s platelet count was still very low (approximately 30 × 10^9^/L) after delivery. According to the PLASMIC scoring system [12], the PLASMIC score of the patient was 6.Although the ADAMTS13 test result was still not available, we decided to initiate plasma exchange immediately. Intravenous methylprednisolone 40 mg/day and intravenous immunoglobulin 20 g/day were started at the same time. As expected, the platelet count started to rise after the first plasma exchange. After daily plasma exchange for 4 days, the platelet count increased to 85 × 10^9^/L (Fig. 1A), the LDH level decreased to 548 U/L, and the LDH/AST ratio decreased to 24 on October 25, 2019 (Fig. 1B). The platelet count finally returned to normal on day 10 after delivery (Fig. 1A). One week later, the ADAMTS13 result because available and indicated that the enzyme activity was 0%.

**Fig. 2:**
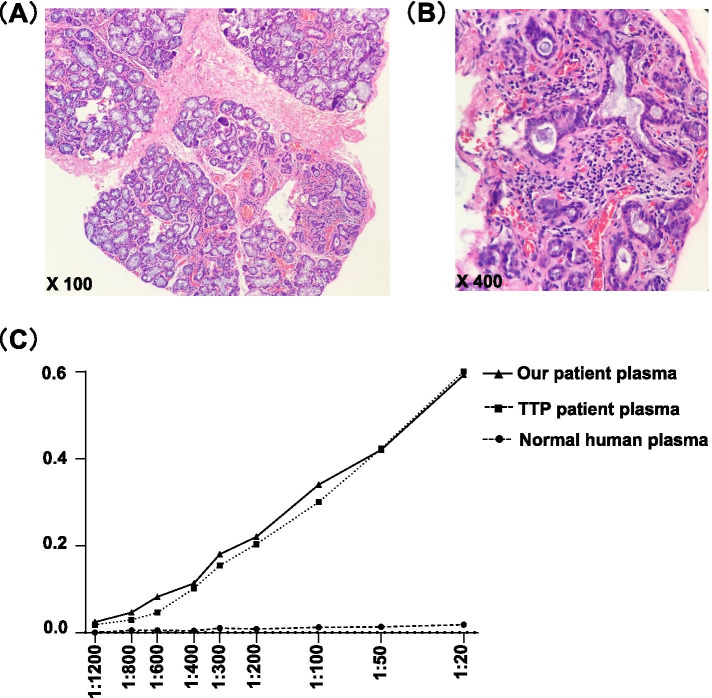
The patient was concomitant with Sjögren’s syndrome harboring nonneutralizing antibodies. (**A** + **B**) Labial gland biopsy of the patient show Sjogren’s syndrome. (**C**) Anti-ADAMTS-13 IgG antibodies detected by ELISA

Gene sequencing of the ADAMTS13 gene and ADAMTS13 inhibitor analysis were performed to clarify whether the patient had cTTP or aTTP. The ADAMTS13 inhibitor assay was performed as described by Yue et al. [13]. Surprisingly, there were no pathogenic mutations in the patient’s ADAMTS13 gene and no inhibitor was detected. At the same time, given that she was anti-SS-A antibody-positive, which led to suspicion of pSS, an ocular and dental sicca evaluation and a labial gland biopsy were also performed. Lacrimal gland and salivary function was found to be normal and the labial gland biopsy showed Sjögren’s syndrome (Fig. 2A, B) that met the diagnostic criteria for pSS [14]. Given that aTTP is usually associated with autoimmune disease [8], the patient was strongly suspected to have aTTP. To confirm the diagnosis, an enzyme-linked immunosorbent assay was performed as described previously [11] and clearly identified the ADAMTS13 IgG antibody (Fig. 2C). Due to the low enzyme activity in this patient, which suggested a high risk of recurrence, we administered rituximab (375 mg/m^2^ qw * 2 w, 100 mg/m^2^ qw * 2 w) as preventive treatment. One month later, the ADAMTS13 enzyme activity increased to 100%, which also pointed to a diagnosis of aTTP. Therefore, a diagnosis of pregnancy-associated aTTP secondary to pSS was finally made. Six months later, the patient’s platelet level had stabilised at a normal level and the activity of the ADAMTS13 enzyme was still 100%. However, her anti-SS-A antibody and ANA were still positive. The patient suffered from occasional arthralgia. She has been treated with hydroxychloroquine 200 mg twice daily, prednisone 5 mg every other day and methotrexate 10 mg once a week until now. The child had a normal development at eighteen-month-old.

## Discussion and conclusions

The differential diagnosis of thrombocytopenia during pregnancy remains a significant clinical challenge because of its heterogeneous clinical presentation, which includes TTP, haemolytic uraemic syndrome, disseminated intravascular coagulation, HELLP syndrome, and immune thrombocytopenia. Generally, it is difficult to make a diagnosis in the early stages of TTP because typical symptoms and signs have yet to become apparent. Misdiagnoses have been noted in several reports [15, 16]. For example, Jonathan et al. [15] described a patient with TTP who was misdiagnosed to have HELLP syndrome at gestational week 37 because she presented with systolic hypertension, proteinuria, and hepatic dysfunction without central nervous system findings or fever. The patient’s platelet level did not increase after receiving high-dose dexamethasone, antihypertensive medication, and platelet transfusion. Three days later, her renal function started to deteriorate markedly, and the patient died soon afterwards. Another case report concerned a 20-year-old woman who developed thrombocytopenia during pregnancy and was misdiagnosed to have immune thrombocytopenia [16]. She developed severe bleeding and fever without haemolysis, renal failure, or neurological symptoms. Bone marrow analysis showed decreased biogenesis of platelets. She also had high blood pressure, headache, and elevated hepatic enzymes and LDH after steroid therapy and splenectomy. Caesarean section was performed at gestational week 27. Fragmented red cells were then found in the patient’s peripheral blood smear. After plasmapheresis, there was a significant increase in her platelet count. These cases suggest that it is difficult to diagnose TTP clinically in the early stage of the disease when there are no obvious clinical manifestations. Analysis of ADAMTS13 is important in the early diagnosis of pregnancy-associated TTP.

In the absence of ADAMTS 13 test result, the PLASMIC score can also be helpful to distinguish TTP from a broad range of thrombotic microangiopathy subtypes [12, 17], It is composed of seven elements: platelet count < 30 × 109/L; combined haemolysis variable (reticulocyte count > 2.5%, or haptoglobin undetectable, or indirect bilirubin > 2.0 mg/dL); absence of active neoplasia; absence of an organ or stem-cell transplant; mean corpuscular value (MCV) < 90 fL; international normalised ratio < 1.5; and creatinine < 2.0 mg/dL [12]. A PLASMIC score of 0–4 denotes low risk, a score of 5 denotes inter mediate risk, and a score of 6 or 7 denotes high risk. High risk recorded in 62–82% of patients with severe ADAMTS13 deficiency [12]. Plasma exchange is recommended for Score ≥ 6. Report shows that a combination of the PLASMIC score of 6–7 and LDH-to-AST ratio of ≥5.5 show a higher positive predictive value for TTP in pregnant patients than the PLASMIC score alone [18].

Pregnancy is a predisposing factor for autoimmune disease [8, 19–21]. Changes in hormone levels may induce autoimmune disease, sometimes causing occult or atypical cases with significant clinical manifestations or triggering relapse or aggravation of disease [20]. Autoimmune diseases can produce a variety of autoantibodies, including ADAMTS13 antibodies, which result in onset of TTP. Moreover, there is an increase in the von Willebrand factor level and a decrease in the ADAMTS13 level in pregnant women, resulting in an increased risk of TTP in pregnancy [22]. Therefore, it is important to identify autoimmune disease early and to implement monitoring during pregnancy to reduce the damage to both mother and child. If patients with connective tissue disease develop thrombocytopenia, we should consider the possibility of TTP and check the ADAMTS13 level.

pSS is an autoimmune disease with a high prevalence of anti-SS-A (anti-Ro) and anti-SS-B (anti-La) antibodies and is known to occur predominantly in women. Women with pSS are likely to have more complicated pregnancies than their counterparts without the disease [23]. Our patient did not have any symptoms of dryness and had not undergone an immune examination before onset of TTP. Asymptomatic pSS was diagnosed incidentally on screening. Therefore, it is unknown whether pSS had occurred before or was induced by pregnancy. Given that there were no other predisposing factors for TTP in this patient, the cause of TTP was considered tobe closely related to her pregnancy and pSS. To the best of our knowledge, only three cases of aTTP combined with pSS have been reported in the past 20 years, and none were associated with pregnancy [24–26]. Our case emphasises the importance of screening for collagen disease during pregnancy, including pSS, even if the patient is asymptomatic.

Notably, in this case, the ADAMTS13 antibody could not be detected by conventional methods but was finally identified by enzyme-linked immunosorbent assay using immobilised recombinant ADAMTS13, which is consistent with the scenarios described in other studies [11, 27]. It is possible that the patient had an abnormal autoimmune system with non-neutralising antibodies that affected the half-life of the ADAMTS13 enzyme or its ability to bind with the surface of endothelial cells, thereby damaging the activity of ADAMTS13 in vivo [11].

The standard of treatment in aTTP has been PEX combined with steroids [4, 28]. In this case, the activity of the ADAMTS13 enzyme remained at 0% for 2 months with normal platelet and haemoglobin levels, even after early treatment with plasma exchange and glucocorticoids, which suggested there was a high risk of recurrence [29]. The International Society on Thrombosis and Haemostasis has made a conditional recommendation to use rituximab outside of pregnancy for asymptomatic aTTP with low plasma ADAMTS13 activity [30]. Rituximab is an anti-CD20 monoclonal antibody that is thought to resolve TTP by depleting the B-cells that produce the ADAMTS13 inhibitor and preventing recurrence of the disease [1]. After treatment with rituximab, the activity of the ADAMTS13 enzyme increased significantly in our patient from 0 to 100% and remained at 100% for approximately 6 months, which greatly reduced the risk of recurrence. The most commonly used regimen for rituximab is 375 mg/m2 weekly for 4 weeks, which based on the dosing for lymphoma [31]. Recent studies suggest that low-dose rituximab (100 mg fixed dose) in combination with PEX for aTTP has clear advantages [32]. The most recent addition to the treatment of aTTP is caplacizumab, an anti-VWF nanobody, which inhibits interaction between VWF multimers and platelets, reducing platelet aggregation and microvascular thrombosis [33, 34]. At present, the safety of caplacizumab in pregnant women is not clear.

This report describes the first patient with pregnancy-associated TTP complicated by pSS who had non-neutralising antibodies and was successfully treated with rituximab in addition to plasma exchange and pulse corticosteroid therapy. We believe that this case is clinically instructive in several aspects, particularly concerning the diagnosis and treatment of TTP. However, an important limitation of our work is that the exact cause of TTP in this complex case is unknown. We supposed that dysimmunity due to asymptomatic Primary Sjögren Syndrome might contribute to the onset of TTP and pregnancy was the trigger for TTP in this autoimmune process. More researches should be done in the future.

## Supplementary Information

**Additional file 1.**

**Additional file 2.**

**Additional file 3.**

## Data Availability

All data analyzed during this study are included in this report and additional supporting files.

## Abbreviations

ANA: Anti-nuclear antibody
aTTP: Acquired thrombotic thrombocytopenic purpura
BP: Blood pressure
cTTP: Congenital thrombotic thrombocytopenic purpura
DIC: Disseminated intravascular coagulation
ELISA: Enzyme-linked immunosorbent assay
HELLP Syndrome: *H*aemolysis *E*levated *L*iver enzymes *L*ow *P*latelets syndrome
HUS: Haemolytic uraemic syndrome
ITP: Immune thrombocytopenia
pSS: Primary Sjögren’s syndrome
SS: Sjögren’s syndrome
TMA: Thrombotic microangiopathy
TPE: Therapeutic plasma exchange
TTP: Thrombotic thrombocytopenic purpura
LDH: Lactate dehydrogenase
AST: Aspartate aminotransferase
