# Supplementary figures and images for: Pregnancy-associated thrombotic thrombocytopenic purpura complicated by Sjögren’s syndrome and non-neutralising antibodies to ADAMTS13: a case report
Source: BMC Pregnancy Childbirth. 2021 Dec 3;21:804. doi: 10.1186/s12884-021-04167-9 (PMC8641216; doi:10.1186/s12884-021-04167-9)

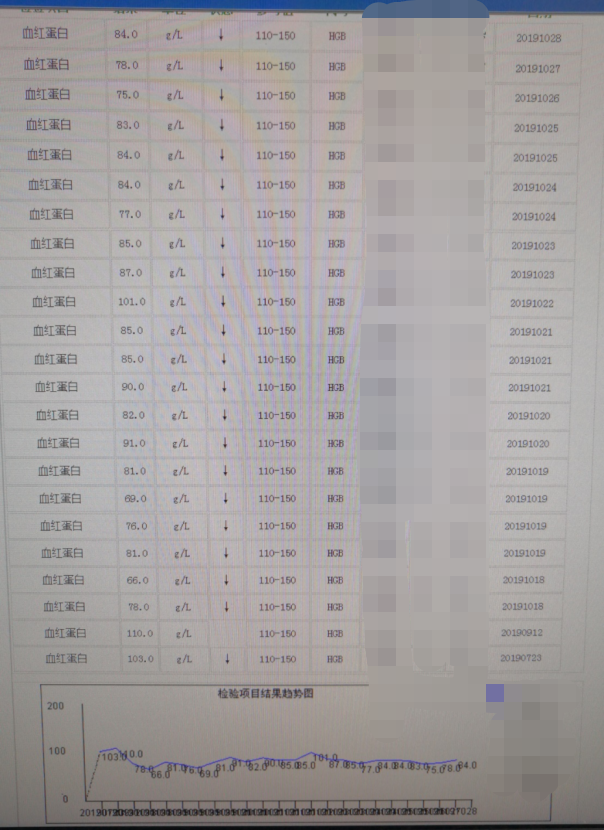


Hemoglobin level


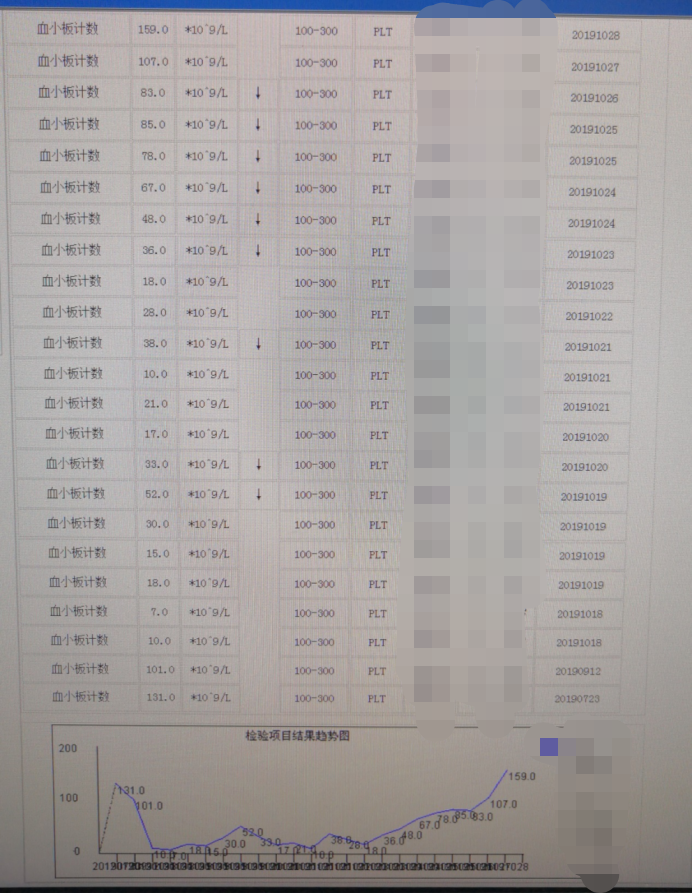


Platelet level


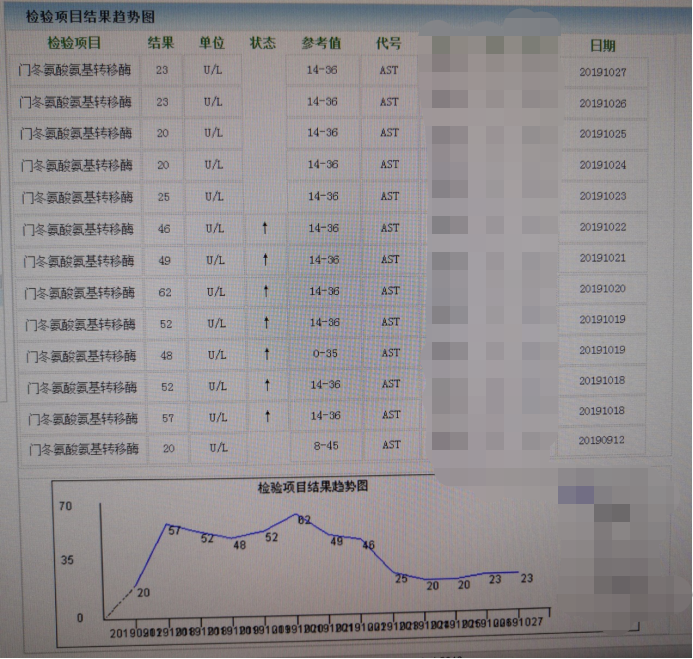


AST level


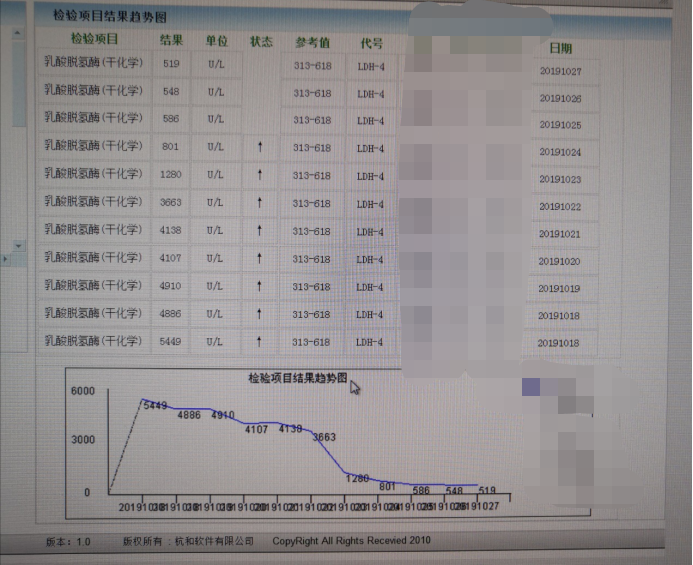


LDH level

Supplement: Supplementary file 2 — Additional file 2. [file 12884_2021_4167_MOESM2_ESM.docx]
